# Validation of an improved reference freeze-dried direct agglutination test for detecting leishmaniasis in the canine reservoir

**DOI:** 10.1099/acmi.0.000890.v4

**Published:** 2025-01-06

**Authors:** Abdallah el Harith, Elfadil Abass, Franjo Martinkovic, Durria Mansour, Hussam Ali Osman

**Affiliations:** 1 Department of Biomedical Research, School of Pharmacy, Ahfad University for Women, Omdurman, Sudan; 2 Department of Clinical Laboratory Science, College of Applied Medical Sciences, Imam Abdulrahman Bin Faisal University, Dammam, Saudi Arabia; 3 Department for Parasitology and Parasitic Diseases with Clinic, Faculty of Veterinary Medicine, University of Zagreb, Zagreb, Croatia; 4 Department of Research and Grants Unit, Ahfad University for Women, Omdurman, Sudan; 5 Department of Medical Laboratory Science, Faculty of Medical and Health Sciences, Liwa College, Abu Dhabi, UAE

**Keywords:** anti-clumping agent, canine leishmaniasis, citrate-saline formaldehyde, detection, freeze-dried direct agglutination, improvement, SDS, urea

## Abstract

**Introduction.** Proper identification and management of post-kala-azar dermal leishmaniasis (PKDL) and canine leishmaniasis (CanL) cases are among the prerequisites to the effective control of visceral leishmaniasis worldwide. Unlike PKDL, CanL still awaits effective improvement because of its cryptic nature, absence of *Leishmania* parasites in lesions or lymph nodes and not complete sensitivity of some diagnostic tools in use. Because of the need for certain skills and equipment, both the liquid direct agglutination test and freeze-dried direct agglutination test (FD-DAT) versions are, in comparison with the indirect immunofluorescence antibody test (IFAT) or enzyme-linked immunosorbent assay (ELISA), practical and feasible diagnostic alternatives.

**Aim.** Validate the performance of an improved FD-DAT to suit routine and large-scale applications in CanL endemic areas.

**Methodology.** Introducing citrate-saline formaldehyde (CSF) as an anti-clumping agent to replace normal saline for antigen reconstitution and drastically, however, eligibly lower the concentration of promastigotes (1.4×10^7^) in comparison with the original FD-DAT reference (>5×10^7^ ml^−1^). To ensure optimal safety, *β*-mercaptoethanol was replaced by urea or SDS as a serum-reducing agent.

**Results.** By improving the procedure for reconstitution of FD-DAT antigen with CSF, a 150% reduction in the test application cost was achieved. Expired test batches (±4 years earlier) were successfully revitalized to full validity. As compared to the 48 h shelf-life time for the original, an FD-DAT batch reconstituted here with CSF maintained stability for ±12 months.

**Conclusions.** The highly concordant results with IFAT and ELISA (one-way ANOVA test, *P*=0.142, homogeneity of variances *P*=0.009) as routine CanL diagnostics further motivate the application of the improved FD-DAT for the detection of the disease in endemic areas.

## Data Summary

No additional data are required to reproduce the results obtained in this work.

## Introduction

Because of assimilation with other various canine disorders and the scarcity or complete absence of the causative *Leishmania* parasite in the lesions formed, the diagnosis of canine leishmaniasis (CanL) still constitutes a major drawback for visceral leishmaniasis (VL) control in Europe, South America and North Africa. Although lymph node and bone marrow aspirations are performed in these endemic areas, failure to demonstrate *Leishmania* amastigotes in CanL cases was frequently reported [1]. The indirect immunofluorescence antibody test (IFAT), even though considered complicated to perform, is applied at the central laboratory level in the endemic areas of Southern and Eastern Europe [2]. Depending on the nature of the antigen used, variable efficacy for CanL diagnosis was reported for the ELISA [3,5].

Employing antigen suspension of trypsin-treated and Coomassie Brilliant Blue-stained *Leishmania donovani* promastigotes in a liquid direct agglutination test (LQ-DAT), highly favourable results were reported for VL diagnosis in East Africa [6,7]. By applying the same antigen to sera from Dutch and German dog populations that returned from stays with their owners in Southern Europe, diagnostic reliabilities for CanL highly comparable to those of the Eastern African VL suspects were reported [8]. In order to achieve better stability during transportation or storage under adverse high temperatures, a freeze-dried direct agglutination test (FD-DAT) version of the test was developed and commercialized in the Netherlands [9,10]. The outcome of evaluation using this improved DAT revealed highly encouraging reliabilities for CanL in Dutch dogs that had overwintered with owners in Southern Europe [9]. However, despite the excellent detection reliability reported, routine application of the FD-DAT was not implemented in the CanL major endemic areas, highly likely due to test infrequent availability, failure to produce the test locally or the importation cost involved. The health and environmental hazards associated with the mandatory use of *β*-mercaptoethanol (β-ME) as a serum-reducing agent in test procedures further formed an additional obstacle [11].

During the past decade, significant progress was made that included the production of the LQ-DAT locally and the introduction of essential improvements to the FD-DAT to ensure optimal safety for VL routine diagnosis in Sudan and elsewhere. Aside from a pronounced reduction in test cost using the valid batches, FD-DAT batches that had expired 7 years earlier were successfully revitalized, contributing to a further lowering of the test expenses [12].

In this study, we intended to assess the performances of an improved valid or expired FD-DAT version in comparison with the original reference, LQ-DAT, IFAT and an ELISA version employing a recombinant antigen for the detection of CanL in an endemic dog population from Croatia.

## Methods

### Ethical consideration

This study was ethically approved by the Ethical Review Committee of Ahfad University for Women on 27 February 2023 (Approval No. UERC09921).

### Valid freeze-dried agglutination test batch

Leish DAT Antigen (Royal Tropical Institute, Amsterdam, Netherlands) (Lot no. 1905; production 2022) in 5 ml vials was used. Following the producer’s instructions, the vials were stored at 4–5 °C or at air-conditioned laboratory temperature (20–23 °C) until needed.

### Expired freeze-dried agglutination test batches

Leish DAT Antigen (Royal Tropical Institute, Amsterdam, Netherlands) (Lot no. 1602; production 2016), expired 4 years earlier, was employed.

**Fig. 1.**
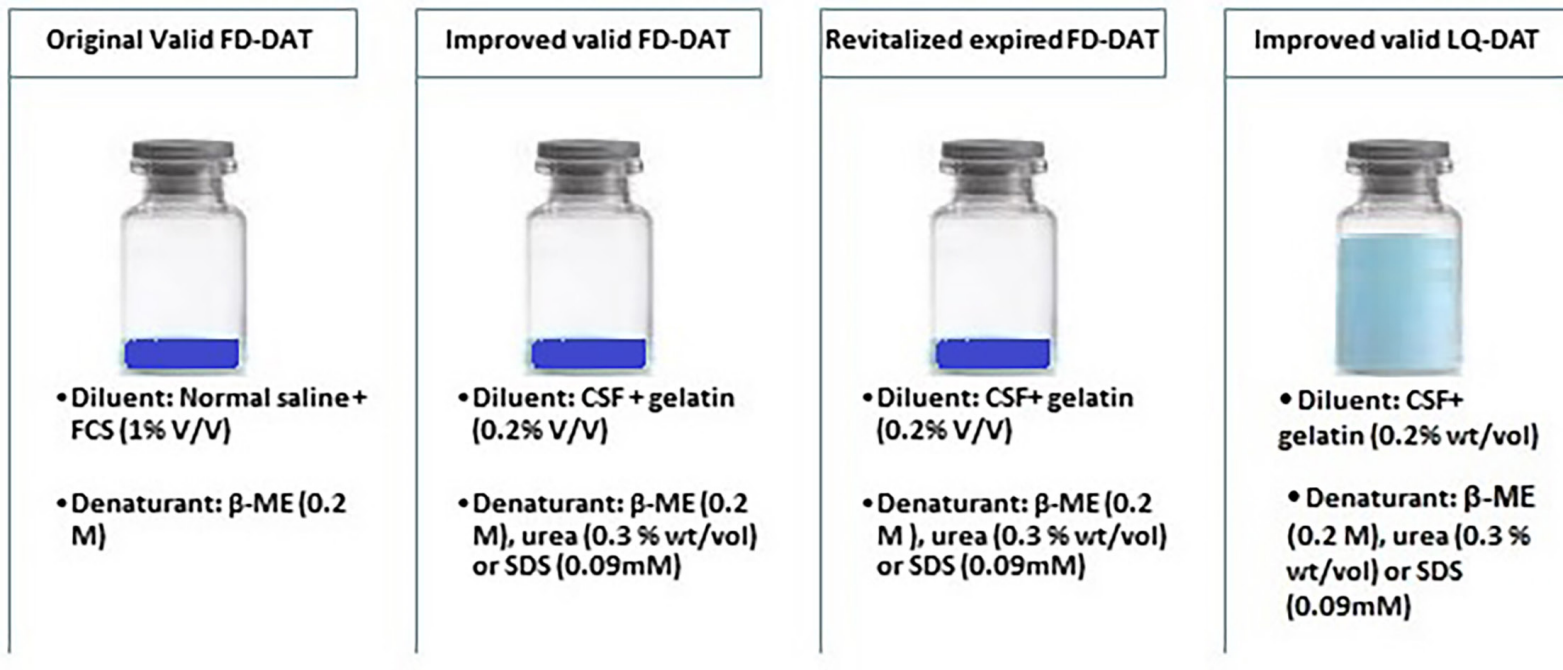
Protocol for the execution of trypsin-, β-ME-, urea- or SDS-modified freeze-dried [freeze-dried direct agglutination test (FD-DAT)] and liquid [liquid direct agglutination test (LQ-DAT)] variants using valid or expired antigen batches. Valid and expired antigens in the improved FD-DAT were reconstituted with CSF. The test sample diluent consisted of normal saline supplemented with FCS and *β*-ME in the execution of the original FD-DAT or in normal saline supplemented with gelatin, *β*-ME, urea or SDS in the execution of both LQ-DAT and improved FD-DAT.

The valid original reference FD-DAT was reconstituted according to the manufacturer’s instructions with 5 ml of normal saline per vial. Following a similar reconstitution procedure with the expired FD-DAT batch resulted in an invalid test outcome. Microscopic examination of samples from both valid and expired FD-DAT batches revealed, except for a reduction in promastigote density/field microscope and the presence of clumps in the expired antigen, comparable parasite morphology in both antigen saline suspensions (Fig. 1).

Therefore, to exclude the possible occurrence of spontaneous agglutination, both valid and expired FD-DAT batches were reconstituted with an anti-clumping solution {0.056 M sodium citrate and 0.15 M sodium chloride supplemented with 1.2% w/v formaldehyde [citrate-saline formaldehyde (CSF)] instead of the normal saline originally instructed}. To further improve feasibility by reducing the concentration of promastigote per unit of antigen suspension to a minimal yet eligibly sufficient level, parasite densities significantly lower than those originally reported (9.0×10^7^ ml^−1^) were tested [12]. The reconstituted valid and expired FD-DAT aliquots were stored at 4 °C until required.

Execution of the original reference FD-DAT was done as instructed using foetal calf serum (FCS, 1% v/v) in normal saline. As a diluent for both, the improved valid or expired FD-DAT, FCS was replaced by gelatin at 0.2% wt/vol. in normal saline (Fig. 1). The gelatin/saline mixture was heated up to 80 °C [7], after which the mixture was left to cool at room temperature. To compare the efficacy to eliminate non-specific agglutination reactions using the original reference or both the improved valid and expired, *β*-ME (0.8% vol./vol.) or urea (0.3% wt/vol.) was used as reducing agents in the FCS or gelatin/saline diluent as described in detail earlier (Fig. 1) [7].

Based on recent observations, the supplementation of SDS (0.045 mM) in a gelatin diluent containing NaCl (0.15 M), CaCl2 (0.02 M), KCl (0.05 M) and NaHCO3 (0.05 M) proved to be highly efficient for the elimination of non-specific agglutination and therefore included in this study as a third reducing agent (Fig. 1) [13].

Execution of the test with the three FD-DAT antigen types (original reference, improved valid or improved expired) was carried out using V-shaped well microtitre plates employing initially single dilution testing at 25 or 100 depending on the objective of the experiment (Table 1). As in our routine testing procedures for VL in human samples, microtitre plates were incubated at an air-conditioned laboratory temperature (20–23 °C) during working hours and 23 °C > 40 °C (without air conditioning) at night due to the limitation of budget without noticeable impact on test validity. After 18 h of incubation, test results were virtually read against a white background. The reaction end-point was estimated by localizing a clear sharp-edged blue spot identical to the one discernible in the control well; the preceding sample dilution was considered the titre of the test serum. In this as well as in all the other three studies with either the FD-DAT valid or expired or LQ-DAT, a titre of 400 was taken as the CanL cutoff.

**Table 1.** Comparative efficacies of *β*-ME, urea and SDS as reducing agents for the elimination of non-specific agglutination in sera of six dogs with negative, marginal or positive test outcomes

FD-DAT	No. of dogs	Serum pretreated with the reducing agent			
		Untreated (standard)*	β-ME	Urea	SDS
Improved valid	2	200, 400	50, 100	25, 50	25, 50
	2	800	3200	3200, 6400	3200
	2	12 800, 51 200	25 600, 204 800	25 600, 204 800	25 600, 204 800
Improved expired	2	200, 400	50	25, 50	25, 50
	2	800	3200	3200	3200
	2	12 800, 51 200	25 600, 204 800	25 600, 204 800	51 200, 204 800
Original reference	2	200, 400	50, 100	50	25, 50
	2	800	1600, 3200	3200, 6400	3200
	2	12 800, 51 200	25 600, 204 800	25 600, 204 800	51 200, 204 800

* 400 as cutoff titre for CanL determined by the original saline FD-DAT in sera with titres <400, 800–6400, or >12 800 classified, respectively, as negative, marginal or positive.

### LQ-DAT

*L. donovani* strain isolated from a VL patient residing in the Gedarif area, Eastern Sudan, was used as a source for antigen processing, following the procedures described previously on several occasions [14]. The prepared antigen was then preserved at a concentration of 1.6×10^7^ promastigotes per ml in citrate-saline solution supplemented with CSF to evade auto-agglutination and simultaneously preserve the morphology of the promastigotes. LQ-DAT was executed according to the improved protocol previously mentioned in detail (Fig. 1) [14].

### rKLO8 ELISA

rKLO8 ELISA was performed as described previously using protein concentrations of 5 ng per well in 0.1 M NaCO_3_ buffer, pH 9.6 [15,16]. The coated plates were washed with PBS-Tween 20 and then blocked with 3% bovine serum albumin in PBS, pH 7.5, for 1 h at room temperature to block non-specific binding. After washing steps with PBS-Tween 20, 50 µl of diluted sera at 800 was added to each well. After another washing step, the plates were incubated with peroxidase-conjugated AffiniPure rabbit antidog IgG (H+L) diluted 10000 (ImmunoResearch Laboratories, USA). Colour development was performed with the addition of hydrogen peroxide and tetramethylbenzidine (R and D Systems, Minneapolis, Minnesota, USA). After 10 min of incubation in the dark, 50 µl of 2 N sulphuric acid was added to each well to stop the reaction. The ODs were measured at 450 nm using a microplate reader (FLUOstar Omega, BMG LABTECH) with >0.12 considered indicative for CanL.

### Indirect immunofluorescence antibody test

*L. infantum* promastigote (MON-1) previously isolated from a Croatian dog was harvested at the log phase of growth and washed three times in PBS. They were then re-suspended in the same buffer at a concentration of 10^7^ promastigotes per ml. In total, 10 µl of the so-prepared promastigote suspension were dispensed into multispot slides. The slides were let to dry, fixed with methanol and then washed for 10 min with PBS. They were either stored at +4 °C until used or directly exposed to sera diluted at twofold concentrations starting with 10 in PBS in a moist chamber at 37 °C for 30 min. Excess diluted sera were then removed, and slides were washed thrice in PBS and dried. After drying, promastigotes were let to react with the conjugate (fluoresceinated) rabbit antidog IgG serum (Serotec) for 30 min at 37 °C and afterwards washed three times in PBS and dried. Samples showing cytoplasmic or membranous fluorescence with promastigotes at dilutions >80 were considered indicative for CanL [2].

### Canine sera

In total, 86 sera from both female and male dogs were included in the study. Twenty-two were from healthy Sudanese police dogs at 3–7 years of age (subjected regularly to veterinary check-up), offered by Captain Hassan, Forensic Affairs of the Ministry of Interior, Khartoum North [14]. This dog group was maintained while not on duty, continuously indoors at the Police Department. The other 64 serum samples were from other groups of dogs collected during routine diagnostics in the well-known CanL endemic area of Dalmatia in Croatia and tested initially with IFAT [2]. As some of the Croatian canine sera were not available in sufficient amounts, it was not possible to conduct all the experiments with all of the 86 sera. The serum samples from both Sudanese and Croatian dog groups were stored at −20 °C.

### Statistical analysis

SPSS computer software version 22 was used to measure the variation between performances of the different tests (results normality distributed) by the one-way ANOVA test and homogeneity by the test of homogeneity of variances.

## Results

At promastigote concentration of 1.4×10^7^ ml^−1^, the powder antigen reconstituted with CSF as an anti-clumping agent in both the improved valid and expired FD-DAT showed sharper blue spots in the negative control wells, implying therefore the absence of auto-agglutination as compared with the reference reconstituted with normal saline at 9.0×10^7^ ml^−1^. Unlike the negatives, the CanL-positive samples presented a diffuse (mat) layer on the surface of the V-shaped wells, indicating agglutination reactions either with the original, the improved valid or expired FD-DAT.

In comparison with *β*-ME, both alternative reducing agents, urea and SDS, performed excellently using the original reference or either of the two improved variants. Generally, all three reducing agents showed a comparable noticeable reduction in the non-specific reactions (200–400 down to 25–100) against the CanL seronegative sera but on the contrary, an increase in the specific CanL seropositives (800–6400 up to 1600–204 800) sera (Table 1).

## Discussion

By taking precautions and further using *β*-ME, clearly negative titres (<100) were recorded for all the 51 sera collected from the Sudanese police dogs (22) and the endemic Croatian dogs (29) against the original reference, the improved valid or expired FD-DAT variant (Table 2). Similar performance for the three FD-DAT variants was also observed in sera of the 15 Croatian dogs from the endemic region that scored clearly positive titres of 6400 or higher (Table 2). At 400 titre cutoff, performances of the improved valid or expired FD-DAT were highly concordant with each other as well as with the original freeze-dried or liquid in all 19 Croatian dogs. Titres <200 were recorded with the three test variants in 13 of them, and positive titres ranging 3200–204 800 were measured with the three FD-DAT variants in all of the remaining six dogs (Table 3).

**Table 2.** Performance of the improved valid or expired freeze-dried agglutination test in comparison with the original reference for the detection of leishmaniasis in sera of 22 apparently healthy Sudanese police dogs (22) and 51 CanL endemic Croatian dogs

Total no. of dogstested (73)*	FD-DAT readings†		
	Improved valid	Improved expiredTitre	Original reference
51	≤100	≤100	≤100
1	400	400	800
1	800	400	1600
1	400	400	1600
1	800	800	1600
1	1600	1600	>6400
1	1600	1600	>3200
1	1600	1600	6400
15	>6400	>6400	>6400

* Either no or insufficient quantities of sera were available for testing of 13 Croatian CanL endemic dogs.

**†*β*-ME was used as the reducing agent.

**Table 3.** Performance of the improved valid and expired freeze-dried agglutination test in comparison with the original reference (FD-DAT), the liquid DAT version (LQ-DAT), indirect immunofluorescence (IFAT), ELISA and the manifestation of leishmaniasis (CanL) symptoms in 19 Croatian endemic dogs

No. of dogs	FD-DAT (titre)			LQ-DAT (titre)	IFAT (titre)	KLO8 ELISA (OD)	CanL symptoms
	Improved valid	Improved expired	Original reference				
9	<100	<100	<100	<100	40	0.03–0.09	Clinically healthy
1	<100	<100	200	200	80	0.03	Clinically healthy
1	<100	<100	<100	<100	40	0.15	Dermatitis
1	<100	<100	<100	<100	40	0.16	Clinically healthy
1	<100	<100	<100	<100	40	0.54	Conjunctivitis
1	3200	6400	6400	6400	320	1.2	Clinically healthy
1	51 200	51 200	25 600	25 600	5120	1.93	No data
1	51 200	102 400	204 800	204 800	10 240	1.89	Clinically healthy
1	25 600	51 200	51 200	51 200	640	1.68	No data
1	204 800	204 800	102 400	102 400	2560	0.4	No data
1	3200	6400	6400	3200	80	1.59	Clinically healthy

Comparable performances were also found between the two improved FD-DAT versions and IFAT. Except for 1 out of 13 dogs that tested positive at a low titre of 80, all other 12 scored distinctive negative titres (<40) in IFAT or of 100 against either of the two improved FD-DAT versions (Table 3). Matching high or medium positive IFAT titres were recorded also with the improved FD-DAT versions in six of the seropositive dogs.

Based on OD, a cutoff value of 0.12, 10 out of the 19 Croatian dogs that tested clearly negative in all four DAT versions also showed negative outcomes with rKLO8 ELISA; two others had very low positive OD values (0.15 or 0.16). All six that showed positive titres in all four DAT versions and in IFAT also had matching rKLO8 ELISA outcomes. In none of the three dogs that tested positive in all four DAT versions, as well as in IFAT and KLO8 ELISA, CanL clinical symptoms were observed (Table 3).

The one-way ANOVA test showed no significant variation between performances of the four DAT versions on the one hand or between the four DAT versions and IFAT or ELISA on the other (*P*=0.142). The test of homogeneity of variances also revealed significant homogeneity between performances of the six serological tests (*P*=0.009).

Due to a lack of absolute specificity, anti-leishmanial administration is sometimes withheld in sick seropositive dogs [4]. Based on highly desirable results obtained through intensive evaluation carried out both in endemic and epidemic situations in Bangladesh and Sudan, the presence of symptoms typifying VL together with positive LQ-DAT outcomes had adequately justified the administration of first-line anti-leishmanials with excellent results [17,18]. Reliable DAT results highly indicative of CanL were also reported in Dutch and German dog populations that had overwintered with their owners in the Mediterranean regions with both LQ-DAT and FD-DAT [8,10]. We think that, due to the unavailability of the test or importation cost involved ($32 per 5 ml vial), no serious attempts were undertaken to assess the merits of applying the DAT as a routine CanL diagnosis, particularly in South America and North Africa. Our objective here is to present some essential improvements introduced to the FD-DAT, which are expected to motivate its application as in the case of VL in the major endemic areas of CanL.

Through the successful replacement of normal saline by formaldehyde/citrate saline (CSF) as an anti-clumping agent for antigen reconstitution and drastically but eligibly lowering promastigote concentration per unit antigen suspension medium, considerable improvement in test feasibility was achieved [12]. A larger volume (12 ml) of an anti-clumping agent CFS could therefore be used in comparison with the 5 ml normal saline in the original reference FD-DAT. A significant lowering in test cost was therefore achieved from $32.0 down to $12.8 per 5 ml vial (Table 4). Further sizeable test cost reduction was achieved by revitalizing test batches to their full validity that were expired 4 years earlier. Therefore, revitalized test batches can be used as valid ones, contributing to further improvement in the test feasibility. Completely different than the original reference FD-DAT, which used normal saline as reconstituent, where a very short shelf-life of 48 h was determined, the CSF reconstituted antigens remained valid for at least 1 year at 4 °C for both the improved valid and expired.

**Table 4.** Improving applicability and feasibility of the reference FD-DAT by replacing the standard normal saline medium with the CSF anti-clumping agent for antigen reconstitution

CSF volumes as multiple of the saline standard (5 ml) for FD-DAT reconstitution*	Lowering promastigote concentration versus a standard of 9.0×10^7^ ml^−1^ in FD-DAT	Increase in the no. of test doses per 5 ml vial	Application cost per 5 ml vial versus the original ($32.0)
1 (100%)	0%	8 (0%)	$32.0 (100%)
1.2 (120%)	13.3%	10 (25%)	$25.6 (80%)
1.4 (149%)	27.8%	11 (37.5%)	$23.3 (72.8%)
1.6 (160%)	40.0%	13 (62.5%)	$19.7 (61.6%)
1.8 (180%)	47.8%	15 (87.5%)	$17.0 (53.1%)
2.0 (200%)	62.2%	17 (112.5%)	$15.0 (46.8%)
2.2 (220%)	74.4%	18 (125.0%)	$14.2 (44.4%)
2.4 (240%)	84.4%	20 (125.0%)	$12.8 (40.0%)
2.6 (260%)	91.1%	nd	nd

* * Revealing respective promastigote concentrations of 9.0×107, 7.8×107, 6.5×107, 5.4×107, 4.7×107, 3.4×107, 2.3×107, 1.4×107 or 0.8×107 ml−1 antigen suspension.

** **nd: not included in the comparison due to invalid test outcome.

The use of a single sample dilution at 100 for the initial screening to help identify potential CanL cases has also allowed for the economical utilizing of the improved valid and expired FD-DAT. Further testing to full-out titration (≥6400) for determining the sample, end-point titre could then be carried out starting at the 400 cutoff titre for CanL.

Possibly because of the presence of non-specific natural antibodies at levels exceeding those in the human host, the use of reducing agents in DAT execution seemed therefore indispensable in canine sera. Unlike LQ-DAT and FD-DAT initial versions, *β*-ME was used as the sole reducing agent [8]. However, because of the associated health hazards and inconvenience in use, reducing agents with minimal or no toxicity such as urea were introduced with success. In this study, we also have considered the possibility of introducing a third reducing agent, namely, the SDS emerging from the highly encouraging results recently observed with human plasma from patients diagnosed with haematological malignancies [13]. As shown in Table 1, all three reducing agents performed satisfactorily in lowering non-specific agglutination reactions in the CanL negatives while showing increased or maintaining levels in those of the specific noticeably enhancing, therefore, specificity of all four DAT versions. Both urea and SDS are by far less toxic, convenient and economical to work with compared to β-ME. In comparison with urea or SDS, *β*-ME use proved also inconvenient due to the offensive odour and the need to follow measures to minimize inhalation. To further reduce application costs and circumvent the necessity for a cooling system in the field, FCS was replaced by gelatine with no negative effect on the test performance [7].

The desirable favourable effect of urea or SDS as compared with *β*-ME was further clearly reflected in the agreeable outcomes using the two improved FD-DAT versions (Table 2). All 51 dog sera that clearly tested negative with the original reference had scored equally low titres (≤100) and all 15 that showed highly positive titres with the original did equally so (≥6400) with both the improved valid and expired FD-DAT versions.

The highly promising performance of these two versions was evidently supported by their concordant outcomes with those of IFAT as well as with an ELISA using a recombinant antigen (rKLO8) in 19 of the endemic Croatian dogs. Although 6 out of those 19 scored clear positive readings in all 3 sero-diagnostic tests used, no typical CanL clinical signs were manifested in any of them (Table 3). This observation was in agreement with other reports, implying that suspicion of CanL cannot solely be based on the appearance of the clinical signs. Considering warnings that treatment failures usually occur when initiated after clinical signs appear, along with the common difficulty in demonstrating the parasite, we believe that, as in the current strategy for VL management, the administration of anti-leishmanials should also be seriously considered for the sick seropositives CanL cases [18,19]. Since both IFAT and ELISA require certain skills and equipment, LQ-DAT or here improved FD-DAT, because of their lower application cost and simplicity in execution, provides excellent practical diagnostic substitutes.

Based on intensive experience gained during the past three decades at both laboratory and field levels in Africa, Asia, Southern Europe and South America, where two species from *L. donovani* group are endemic, we strongly believe that through the use of the LQ-DAT or FD-DAT here adequately optimized, and by following a flexible treatment strategy of sick seropositive dogs, a significant reduction in CanL prevalence can be achieved [17,20].

Through further establishing regular surveillance programmes, active disease detection, treatment and follow-ups of detected seropositive dogs [1], using any of these two DAT versions, we think that a more effective strategy for CanL control can be achieved. Combined with preventive measures, including the use of long-lasting insecticides and repellents in spray, spot-on formulation or collar form, and encouraging the use of available purified or recombinant *L. infantum* fraction vaccines, even better results can be achieved [21,22].

## Abbreviations

CanL: canine leishmaniasis
CSF: citrate-saline formaldehyde
FCS: foetal calf serum
FD-DAT: freeze-dried direct agglutination test
IFAT: indirect immunofluorescence antibody test
LQ-DAT: liquid direct agglutination test
ß-ME: ß-mercaptoethanol
VL: visceral leishmaniasis
